# Case report: Inspiration from a rare fatal heart perforation after percutaneous vertebroplasty

**DOI:** 10.3389/fsurg.2023.1227056

**Published:** 2023-09-04

**Authors:** Ke Deng, Jia-Lin Yu, Ye-Jun Feng, Kui Huang, Guo-Feng Wu

**Affiliations:** ^1^ Department of Orthopaedics, The First Affiliated Hospital of Yangtze University, Jingzhou, China; ^2^ Department of Orthopedics, South University of Science and Technology Hospital, Shenzhen, China

**Keywords:** percutaneous vertebroplasty, intracardiac cement embolism, bone cement leakage, osteoporosis, vertebral compression fracture

## Abstract

The principal benefit of employing percutaneous vertebroplasty (PVP) for managing osteoporotic vertebral compression fractures lies in its capacity to facilitate early mobilization in elderly patients, thereby effectively avoiding the potential catastrophic complications associated with prolonged bedridden states. However, bone cement leakage, as the most common complication of PVP, may have fatal consequences. Here, we report a case involving an 85-year-old male patient with L1 vertebral compression fracture who underwent PVP at our hospital and was discharged on the same day of the surgical intervention. Subsequently, the patient experienced symptoms of chest tightness and palpitations. Cardiac ultrasound examination revealed pericardial effusion, while pulmonary computed tomographic angiography (CTA) demonstrated a strip high-density shadow in the right ventricular area. Finally, it was determined that the perforation of the right ventricular wall was caused by bone cement embolism. Through this comprehensive case report, we aim to deepen the understanding of orthopedic doctors on the importance of preventing bone cement leakage.

## 1. Introduction

Percutaneous vertebroplasty (PVP) is a minimally invasive surgical procedure primarily utilized for the treatment of osteoporotic and osteolytic fractures resulting from various etiologies (1). By injecting bone cement into the vertebral body, PVP effectively stabilizes the bone and provides pain relief. Early intervention with PVP is crucial for patients to minimize complications associated with prolonged bedridden states (2). Despite being a minimally invasive operation, PVP is susceptible to bone cement leakage, posing potential risks to patients. Intracardiac cement embolism (ICE) is an uncommon but potentially catastrophic complication of PVP. While most instances of leakage are asymptomatic in clinical settings, the penetration of free bone cement into the heart can result in pericardial tamponade, posing a life-threatening situation (3). In this report, we present a rare case of heart rupture caused by bone cement following PVP, underscoring the significance of recognizing and managing complications associated with this procedure. Our aim is to raise awareness among orthopedic physicians about potential risks and to get valuable experience from this particular case to prevent and manage similar occurrences in the future.

## 2. Case report

**Figure 1:**
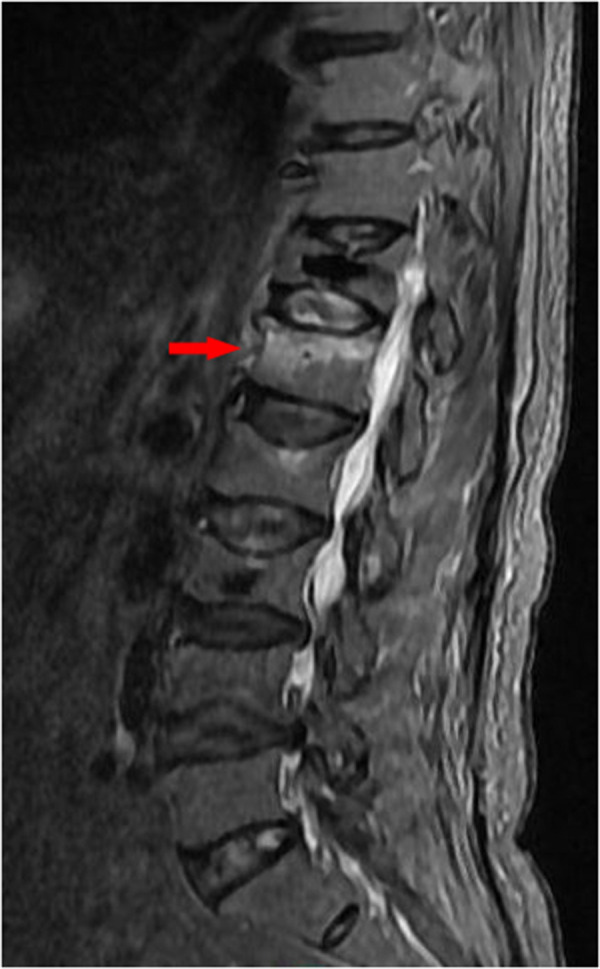
Preoperative MRI of lumbar spine suggested compression fracture of L1 vertebral body.

An 85-year-old male patient was admitted to the hospital because of “low back pain with limited activity for 20 days”. The patient's height 172 cm, weight 64 kg, BMI 21.6. The patient had a history of hypertension, denied a history of coronary heart disease, and had a history of thoracolumbar 12, lumbar 4, and lumbar 5 vertebroplasty. Lumbar magnetic resonance examination (Figure 1) was performed on admission. The diagnosis was fresh compression fracture of lumbar 1 vertebral body. Percutaneous vertebroplasty of lumbar 1 vertebral body was performed. The surgical process is as follows: first, locate the lumbar 1 vertebral body, apply local anesthesia at the insertion point, puncture, and fluoroscopy show that the working channel enters the anterior one-third of the lumbar 1 vertebral body along the pedicle of the vertebral arch. Mix bone cement to a wire drawn shape, inject 2.5 ml of bone cement into the right channel to the vertebral body, and the fluoroscopy shows that the bone cement diffuses to the left side of the vertebral body. After the bone cement solidifies, pull out the working channel and perform another fluoroscopy to see that the bone cement is located inside the vertebral body, suturing the skin. The surgery is over. After operation, we provide patients with treatment such as electrolyte supplementation, pain relief, and anti-osteoporosis. The lumbar pain was significantly relieved, and the patient immediately walked down and asked to be discharged.

**Figure 2:**
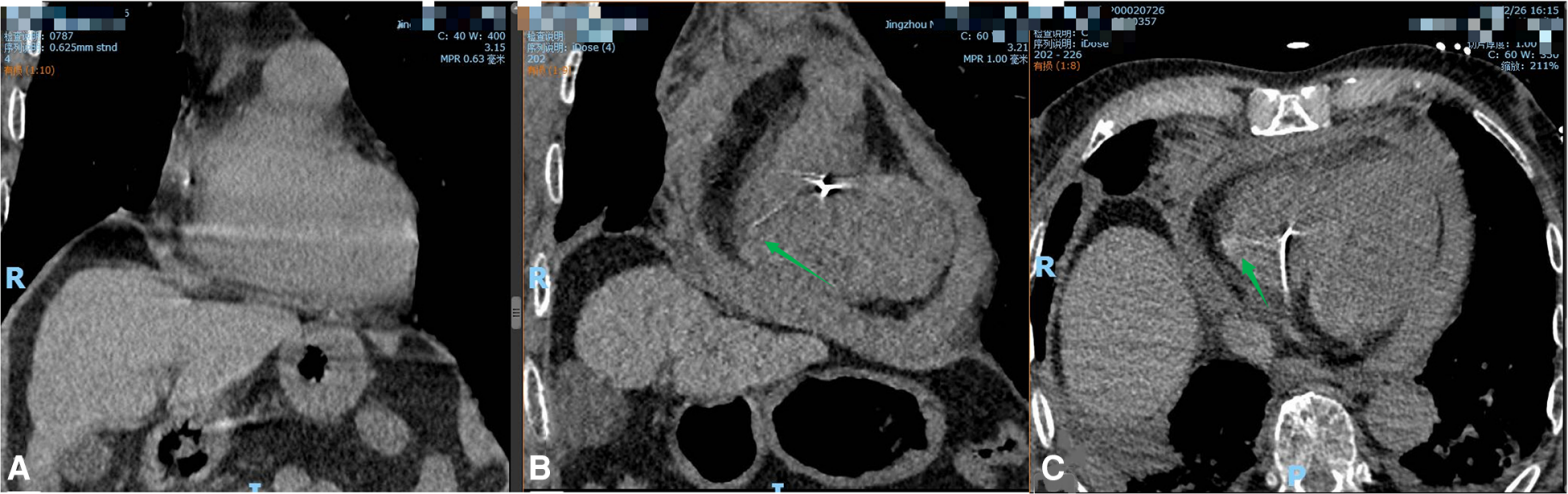
(**A**) Preoperative chest CT coronal plane; (**B**) postoperative chest CT coronal plane showed linear high-density shadow piercing the right ventricular wall, as shown by the arrow; (**C**) after operation, a linear high-density shadow punctured the right ventricle on the chest CT cross section, as shown by the arrow.

Chest tightness and palpitations began to appear on the first day after discharge, and they were sent to hospital again to consider hospitalization for “acute coronary syndrome”. Cardiac troponin and D-dimer were elevated at admission. Cardiac ultrasound showed pericardial effusion. Pulmonary CTA showed strip high-density shadow in the right ventricular area (Figure 2). Cardiac rupture and pericardial tamponade were considered. Subsequently, the patient developed shock and underwent bedside pericardial puncture and drainage and rehydration treatment. The patient's blood pressure returned to normal, and his consciousness recovered.

**Figure 3:**
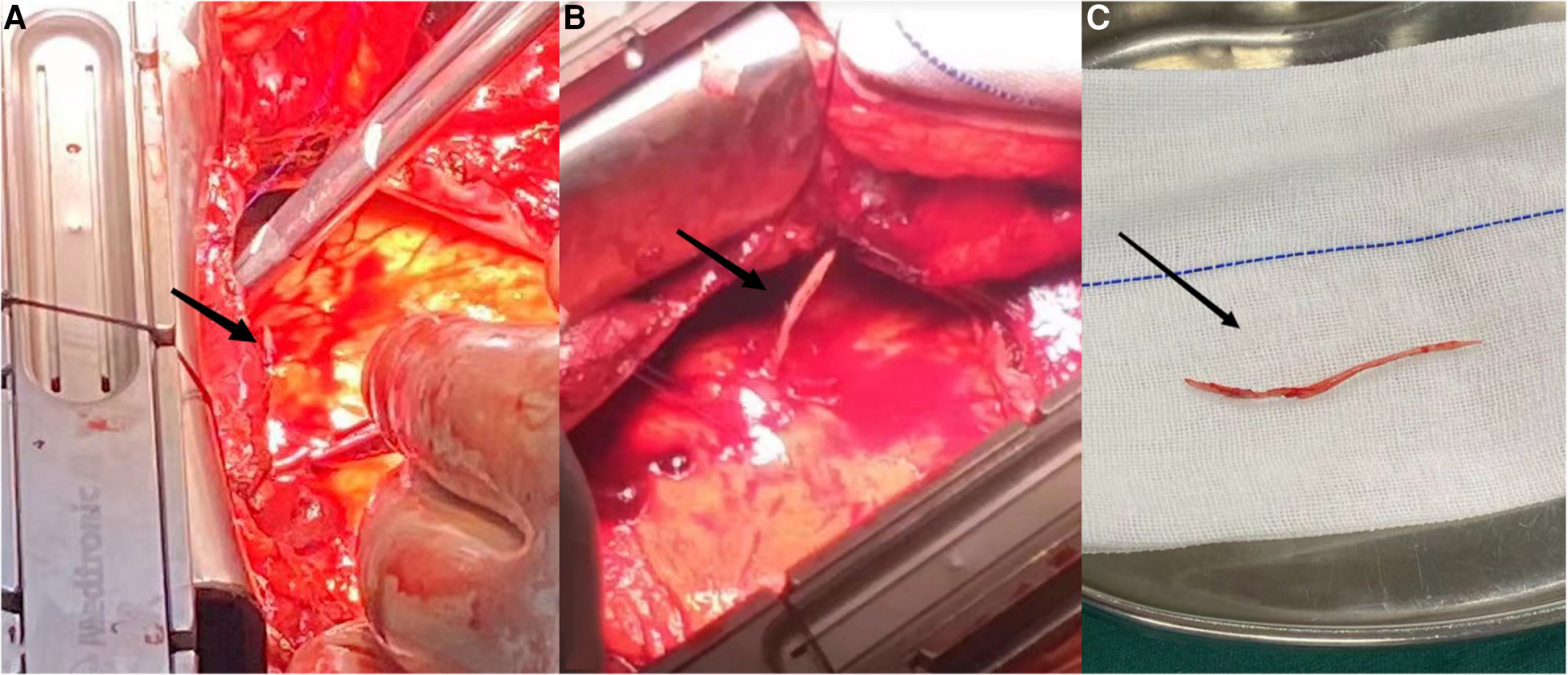
(**A**) Right ventricular linear high-density bone cement punctures the heart; (**B,C**) remove the intracardiac bone cement.

CTA of the patient's pulmonary artery showed a strip-shaped high-density shadow in the right ventricular area, and there was no recent history of trauma and collision. Ice punctured the right ventricular wall and caused cardiac perforation. So, he was transferred to cardiac surgery and underwent thoracotomy under general anesthesia. During the operation, the bone cement tip was exposed from the heart surface and penetrated the right ventricular myometrium. The foreign body was pulled out after purse string suture. The bone cement was strip-shaped, hard, about 8 cm long and about 0.3 cm in diameter (Figure 3) and was transferred to intensive care unit (ICU) for further treatment after operation. Multiple organ failure occurred on the second day after thoracotomy, and death occurred on the 10th day after surgery.

## 3. Discussion

PVP has emerged as the mainstay treatment for osteoporotic vertebral fractures in the elderly, offering the advantages of swift pain relief and early mobilization. However, like any medical procedure, PVP is not without its complications, which include bone cement leakage, nerve function injury, and infection (4). Among them, bone cement leakage is the most common, and although most patients with bone cement leakage are usually asymptomatic, these leakage increase the risk of cardiopulmonary embolism and nerve injury (5). While rare, the occurrence of ICE after PVP is a potentially fatal complication. Although a few reported cases have linked bone cement leakage to cardiac embolism following PVP surgery (6, 7). However, the importance of closely monitoring the position of bone cement during and after surgery has not been adequately emphasized.

Indeed, the prevention of bone cement leakage is paramount in ensuring the safety and success of PVP. The causes of bone cement leakage after PVP can be broadly categorized into three main types: first, insufficient polymerization of bone cement. The operator may adjust the ratio of monomer to powder during surgery to control the viscosity and curing time of the bone cement. The improper ratio of monomer to powder will affect the properties of the material (8). The higher the viscosity of bone cement, the less likely it is to leak (9). Second, the location of the puncture needle. The more the puncture needle pierces the vertebral cortex or the closer the puncture needle is to the vertebral cortex fracture site, the more likely the bone cement is to leak from the damaged part of the vertebral body. The bone cortex cracks can provide space for the cement leakage, especially for patients undergoing multi-level vertebroplasty or patients with highly variable anatomical positions of paravertebral vessels, the more likely it is to leak to the paravertebral vein (10, 11). Third, the bone cement in the vertebral body is overfilled, and the pressure in the vertebral body increases, leading to the leakage of bone cement (12). In addition to these primary causes, factors such as age, preoperative fracture severity, multisegmented fractures, and intravertebral vacuum fractures are closely related to paravertebral bone cement leakage (13, 14). The following measures can be taken to prevent bone cement leakage, including a thorough preoperative evaluation of vertebral body damage, appropriate degree of bone cement solidification during surgery (15), rigorous monitoring during surgery, and appropriate amount of bone cement injection.

## 4. Conclusion

Through the study of the above cases, we realized the importance of preventing bone cement leakage. It is recommended to promptly review and confirm the position of bone cement during and after surgery, and closely observe the changes in the patient's condition after surgery.

## Data Availability

The raw data supporting the conclusions of this article will be made available by the authors, without undue reservation.
